# Adenosine Diphosphate Improves Wound Healing in Diabetic Mice Through P2Y_12_ Receptor Activation

**DOI:** 10.3389/fimmu.2021.651740

**Published:** 2021-03-22

**Authors:** Paula Alvarenga Borges, Ingrid Waclawiak, Janaína Lima Georgii, Vanderlei da Silva Fraga-Junior, Janaína Figueiredo Barros, Felipe Simões Lemos, Thaís Russo-Abrahão, Elvira Maria Saraiva, Christina M. Takiya, Robson Coutinho-Silva, Carmen Penido, Claudia Mermelstein, José Roberto Meyer-Fernandes, Fábio B. Canto, Josiane Sabbadini Neves, Paulo A. Melo, Claudio Canetti, Claudia Farias Benjamim

**Affiliations:** ^1^ Institute of Biomedical Sciences, Center of Health Sciences, Federal University of Rio de Janeiro (UFRJ), Rio de Janeiro, Brazil; ^2^ Fluminense Federal Institute (IFF), Rio de Janeiro, Brazil; ^3^ Institute of Biophysics Carlos Chagas Filho (IBCCF), Center of Health Sciences, UFRJ, Rio de Janeiro, Brazil; ^4^ Institute of Medical Biochemistry Leopoldo de Meis, Center of Health Sciences, UFRJ, Rio de Janeiro, Brazil; ^5^ Institute of Microbiology Paulo de Góes, Center of Health Sciences, UFRJ, Rio de Janeiro, Brazil; ^6^ Center for Technological Development in Health, Oswaldo Cruz Foundation, Rio de Janeiro, Brazil; ^7^ Laboratory of Applied Pharmacology, Institute of Drug Technology, Farmanguinhos, Oswaldo Cruz Foundation, Rio de Janeiro, Brazil; ^8^ Department of Immunobiology, Institute of Biology, Fluminense Federal University (UFF), Niterói, Brazil

**Keywords:** adenosine diphosphate (ADP), wound healing, mice, skin, diabetes, inflammation, purinergic signaling, P2Y_12_ recepor

## Abstract

Chronic wounds are a public health problem worldwide, especially those related to diabetes. Besides being an enormous burden to patients, it challenges wound care professionals and causes a great financial cost to health system. Considering the absence of effective treatments for chronic wounds, our aim was to better understand the pathophysiology of tissue repair in diabetes in order to find alternative strategies to accelerate wound healing. Nucleotides have been described as extracellular signaling molecules in different inflammatory processes, including tissue repair. Adenosine-5’-diphosphate (ADP) plays important roles in vascular and cellular response and is immediately released after tissue injury, mainly from platelets. However, despite the well described effect on platelet aggregation during inflammation and injury, little is known about the role of ADP on the multiple steps of tissue repair, particularly in skin wounds. Therefore, we used the full-thickness excisional wound model to evaluate the effect of local ADP application in wounds of diabetic mice. ADP accelerated cutaneous wound healing, improved new tissue formation, and increased both collagen deposition and transforming growth factor-β (TGF-β) production in the wound. These effects were mediated by P2Y_12_ receptor activation since they were inhibited by Clopidogrel (Clop) treatment, a P2Y_12_ receptor antagonist. Furthermore, P2Y_1_ receptor antagonist also blocked ADP-induced wound closure until day 7, suggesting its involvement early in repair process. Interestingly, ADP treatment increased the expression of P2Y_12_ and P2Y_1_ receptors in the wound. In parallel, ADP reduced reactive oxygen species (ROS) formation and tumor necrosis factor-α (TNF-α) levels, while increased IL-13 levels in the skin. Also, ADP increased the counts of neutrophils, eosinophils, mast cells, and gamma delta (γδ) T cells (Vγ4^+^ and Vγ5^+^ cells subtypes of γδ^+^ T cells), although reduced regulatory T (Tregs) cells in the lesion. In accordance, ADP increased fibroblast proliferation and migration, myofibroblast differentiation, and keratinocyte proliferation. In conclusion, we provide strong evidence that ADP acts as a pro-resolution mediator in diabetes-associated skin wounds and is a promising intervention target for this worldwide problem.

## Introduction

Wound healing is a complex, dynamic and multi-mediated process characterized by a highly regulated cascade of events requiring the interaction of many cell types, including inflammatory and immune cells. Normal healing process occurs over a range of overlapping events: inflammation, granulation tissue formation, and remodeling. Impaired wounds are often associated with pathologic inflammation due to a persistent, incomplete, or uncoordinated healing process (1, 2).

Patients suffer from abnormalities of wound healing worldwide; in particular, under conditions such as senescence, diabetes, ischemia, peripheral vascular disease, and cancer (3, 4). Chronic wounds are reported to affect around 8.2 million patients just in the USA, based on a 2018 retrospective analysis of Medicare beneficiaries; the estimated annual cost for healthcare system to treat wound-related complications is more than US$ 28 billion (5, 6). In Brazil, the most populous country in Latin America, about 40 to 60% of non-traumatic lower limb amputations occur in diabetic patients, whereas, about 85% are related to foot ulcers (7–9).

Among inflammatory mediators, nucleotides play important roles in host defense and tissue repair; however, little is known about their role in wounds (10). The nucleotide adenosine-5’-diphosphate (ADP) plays a pivotal role in the physiologic process of hemostasis and platelet aggregation. ADP activates P2Y_1_, P2Y_12_, and P2Y_13_ receptors, which are expressed by monocytes, macrophages, lymphocytes, mast cells, fibroblasts, keratinocytes, endothelial cells, eosinophils, platelets, neutrophils, and dendritic cells (11–13). Neuroprotective function for ADP was demonstrated in zebrafish retina since it mitigates the excessive cell death and tissue damage; additionally, it stimulated cellular proliferation after injury (14). In addition, ADP induced the proliferation of mouse fibroblasts (3T3 and 3T6), suggesting a positive effect on wound healing (15). Since purinergic system has been involved in pro-inflammatory, cell proliferative and pro-resolution effects, we aim to explore the role of ADP in accelerating wound healing in diabetic mice, considering that chronic wounds are a relevant health problem evidenced by the lack of an effective treatment, especially in diabetic patients.

## Materials and Methods

### Mice

Male Swiss (8-10 weeks) and C57BL/6 mice (10-14 weeks) weighing 25-30 g obtained from the Institute of Science and Technology in Bio Models at Oswaldo Cruz Foundation, were used for full-thickness excisional wound models. For the cutaneous leishmaniasis lesion model, we used male BALB/c mice (10-14 weeks), obtained from the Microbiology and Parasitology Department animal facility at Biomedical Institute in Fluminense Federal University. All procedures described were approved by the Ethics Committee for the Use of Animals of the Federal University of Rio de Janeiro (CEUA/UFRJ: 093/15 and IMPPG 128/15).

### Induction of Diabetes Mellitus

Diabetes was induced by alloxan (65 mg/kg, i.v. in 100 μL of saline) in mice fasted for 12 h (16, 17). Non-diabetic mice were injected with saline (100 μL). Diabetes was confirmed 7 days later when blood glucose concentration was at least 350 mg/dl. The glucose levels were still elevated (over 350 mg/dl) at day 30 after alloxan injection.

### Full-Thickness Excisional Wound Model

At day 7 after alloxan or saline administration, mice were intraperitoneally (i.p. - 10 μL/10 g) anesthetized (ketamine 112 mg/kg and xylazine 7.5 mg/kg) and a full-thickness excisional wound (10 mm in diameter) was executed on the dorsum using biopsy punch. Wounds were treated once a day for 14 days (or until sample collection) with topical application by a micropipette of adenosine-5’-monophosphate (AMP), ADP, adenosine-5’-triphosphate (ATP), adenosine, or pyrophosphate (Sigma-Aldrich, St Louis, MO) at 30 μM (30 μL/mouse - 15.2 µg/kg), or vehicle (30 μL of saline/mouse). In another set of experiments, a dose-response curve of ADP at 30 µM (15.2 µg/kg), 100 µM (51.2 µg/kg) or 300 µM (153.6 µg/kg), administrated topically in 30 μL, was performed. After the application on the wound, mice were placed alone under a glass funnel for 5 min until the solution was absorbed.

### Wound Area Quantification

To determine the wound closure rate, the wound area was evaluated at days 0, 3, 7, 10, and 14 after wounding. Photographs were taken at a standard distance using a tripod and were analyzed using ImageJ software (National Institutes of Health – NIH). Data were expressed as a percentage of the initial wound area.

### Treatments

The administration of Clopidogrel^®^ (Clop - 5 mg/kg) was performed daily by oral gavage, 1 h before ADP administration for 14 days. Antagonists of P2Y_1_ (MRS 2179 - 30 µM/30 μL/mouse - Tocris, Bioscience, UK) and P2Y_12_ (MRS 2395 - 30 µM/30 μL/mouse - Sigma-Aldrich) receptors, and ATP diphosphohydrolase (apyrase - 6 U/mL, 30 μL/mouse - Sigma-Aldrich – A6535-100UN) were topically applied for 14 days, 30 min before ADP administration. The apyrase used in our work was purified from potato and has predominantly the low ATPase/ADPase ratio of ~1:1 (Sigma-Aldrich).

### Hematoxylin & Eosin Staining and Total Collagen Quantification

Wound tissues were paraffin-embedded and cut in 5-μm thick sections. Hematoxylin and eosin staining was performed as described elsewhere. Skin sample sections (7-μm) were stained with Picro Sirius Red for total amount of collagen, as previously reported (18). The quantification was determined by morphometric analysis using a quantitative imaging software (ImagePro Plus, version 4.5.1). The percentage of collagen per field was obtained by dividing the total area by the fibrosis area.

### Ecto-Nucleotidase Activity

Ecto-nucleotidase activity was determined in wound homogenates by the rate of inorganic phosphate (Pi) released using the malachite green reactions, as previously described (19). The concentration of Pi released in the reaction was determined by a Pi standard curve and expressed as nucleotidase activity (nmol Pi x h^-1^ x mg ptn^-1^).

### Immunohistochemistry (IHC)

Wound samples collected at day 7 were paraffin-embedded, cut in sections (7-μm) and immunostained for several markers, as described previously (20). The specific markers are detailed in the **Supplementary Material**. Data were expressed as number of positive cells per field. For collagen type markers, we employed a score method described by Calvi et al. (21), for the semi-quantification of collagen deposits performed by two different observers, as reported in the **Supplementary Material**.

### Immunofluorescence

Wound sections (5-μm) were immunostained against α-smooth muscle actin (α-SMA; A-2547, 1:200, Sigma-Aldrich) as previously reported (22). Control experiments with no primary antibodies showed only faint background staining (data not shown).

### Fibroblast Purification and Proliferation Assay

Primary neonate dermal fibroblasts were purified from the abdomen and dorsal skin of C57BL/6 male mice as previously described, with few modifications (23). Briefly, the skin was cut into small pieces and digested with 0.1% dispase (Roche, Mannheim, Germany) at 4°C for 24 h. After removal of the epidermal layers, the remaining dermal parts were incubated with 0.1% collagenase D (Sigma-Aldrich) at 37°C for 1 h. Next, the digested cells were passed through a 40-μm cell strainer. Fibroblasts (2 x 10^4^ cells) were cultured for 5-bromo-2’-deoxyuridine (BrdU) staining proliferation assay, as previously described (24). The images were captured using a fluorescence microscope and analyzed using ImageJ software. Results were expressed as the percentage of BrdU^+^ cells by total number of cells labeled with 4’,6-diamidino-2-phenylindole (DAPI).

### Wound Scratch Assay

Primary dermal fibroblasts were seeded and grown until 90% confluence to evaluate migration-induced effect of ADP (10, 30, or 100 µM), as previously reported (25). Pictures of the scratched areas were taken at 0, 6, 12, 18, and 24 h using an inverted microscope equipped with a digital camera (BEL Engineering - Monza, Italy). The areas were measured using the ImageJ software and the fibroblast migration was expressed as % of open area compared to the initial area (0 h – 100%).

### Flow Cytometry

Flow cytometry of the wound tissues was performed as previously described (26). Briefly, wound tissues were digested by an enzyme cocktail (reported in **Supplementary Material**) and the cells (10^6^ cells/mL) were subjected to flow cytometry procedure, stained, and analyzed. Lymphocyte populations recovered from skin and draining inguinal, axillary, and brachial lymph nodes were also analyzed. For skin regulatory T (Tregs) cells analysis, samples were enriched by Percoll gradient for mononuclear cells. Samples were acquired with BD FACS Canto II (BD Biosciences, San Jose, CA) and then analyzed with FlowJo software. Gating strategy and the list of antibodies are described in the **Supplementary Material**.

### Eosinophil and mast cell infiltrates

Skin sections (5-μm) were stained with modified Sirius Red or Alcian Blue for eosinophils and mast cells, respectively, as described elsewhere (27, 28). Images were taken using a digital camera coupled to the microscope (Olympus BX53) at 40x magnification. Twenty fields were analyzed per wound/animal (n=3) and the data were expressed as number of eosinophils or mast cells/mm^2^.

### Myeloperoxidase Activity

The number of neutrophils was indirectly determined by myeloperoxidase enzyme activity in the wounds removed 7 days after wounding, as previously described (29). The number of neutrophil was estimated by a standard curve, using neutrophils obtained 6 h after i.p. administration of 3% thioglycolate (>90% of neutrophils). Total protein extract was quantified by the Bradford method. Results were expressed as number of neutrophils/mg of protein.

### ELISA

Cytokine quantification was performed in protein extracts from wounds obtained at day 3 and 7 after wounding using PeproTech kits following manufacturer’s instructions. The results were expressed as pg or ng of cytokine/mg of protein.

### Superoxide Assay

The superoxide production assay was performed by the nitroblue tetrazolium (NBT) reaction with reactive oxygen species resulting in formazan as final product (30). Briefly, the wounds were removed at day 7 and homogenized in phosphate buffer saline (PBS) containing protease inhibitors. The formazan formed was measured by ELISA plate reader (620 nm, Spectra Max-250, Molecular Devices). Results were expressed as µg of formazan/mg of protein.

### Cytometric Bead Array (CBA)

Cytokine concentration in the wounds was determined by flow cytometry using the kit CBA Mouse Inflammation (BD Biosciences, San Diego, CA), following manufacturer’s instructions. This CBA kit allows measurements of interleukin-6 (IL-6), IL-10, C-C motif chemokine ligand 2 (CCL2), interferon-γ (IFN-γ), TNF-α, and IL-12p70. Sample processing and data analysis were acquired by FACS Calibur flow cytometer (BD Bioscences) and FCAP Array software, respectively. Results were expressed as pg or ng of cytokine/mg of protein.

### Western Blotting (WB)

Wound homogenates (30 mg of skin tissue – 20 μg of protein loaded per gel lane) collected at day 7 were prepared as previously described (31). Immunoreactive bands for α-SMA (1:1000 - Sigma-Aldrich) and β-actin (1:1000 - Cell Signaling, Danvers, MA) were visualized using an enhanced chemiluminescence reagent (Amersham ECL, Biosciences) and pictures were recorded using Healthcare ImageQuant LAS 4000 (GE Healthcare Life Sciences). Densitometry analysis was performed using ImageJ software and the results were expressed as the ratio of α-SMA/β-actin (housekeeping).

### Statistical Analysis

Statistical differences in the wound closure experiments were determined using two-way ANOVA followed by Bonferroni post-test. The significance of other experiments was determined by one-way ANOVA followed by Tukey post-test or unpaired Student’s t test, as stated in the legends. The statistical tests were performed by GraphPad Prism software.

A more detailed description can be found in **Supplementary Material**.

## Results

### ADP Improves Wound Healing in Diabetic Mice

**Figure 1:**
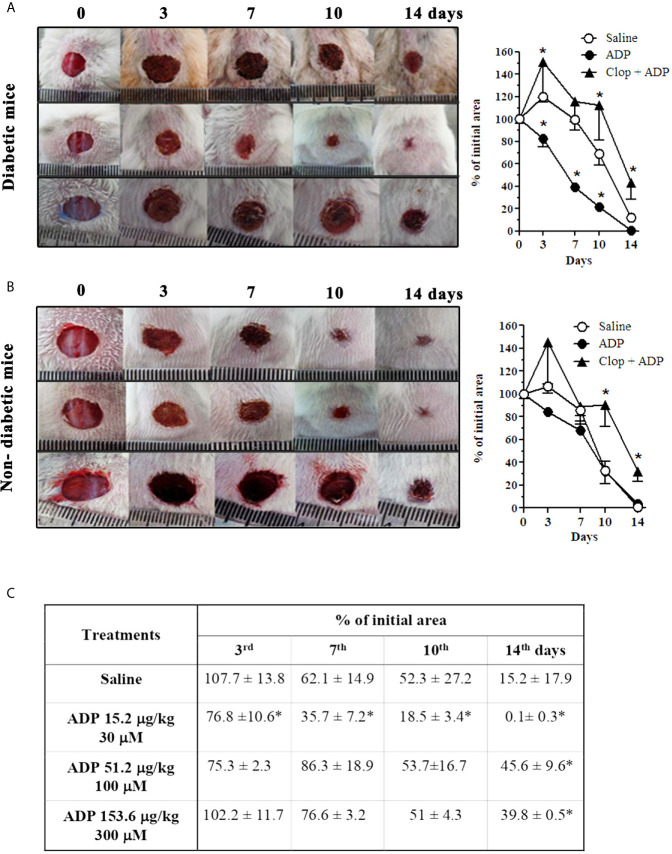
ADP accelerates wound healing in diabetic mice *via* P2Y_12_. Representative images and graphs of diabetic **(A)** and non-diabetic **(B)** mice that were subjected to excisional full-thickness wounding, and then, topically treated with ADP 30 μM (30 μL - 15.4 µg/kg) or saline every day for 14 days. One group of mice was treated by gavage with Clop (5 mg/kg) 1 h before ADP and saline, both once a day for 14 days. Open wound area was measured at days 0, 3, 7, 10 and 14. The areas at day 0 were considered 100%, and the subsequent areas measured at different time-points were calculated as percentages (%) of the initial value. **(C)** Dose-effect data of ADP treatment followed at days 3, 7, 10, and 14 after wounding. Data are expressed as mean ± standard error of the mean. *P < 0.05 by two-way ANOVA followed by Bonferroni post-test, compared to saline-treated mice; n=7-10 per group. Panels A and B are representative of three or more experiments; panel **(C)** represents one experiment.

Diabetic and non-diabetic male Swiss mice were topically treated with saline or ADP 30 μM (30 μL - 15.4 µg/kg), every day for 14 days after wounding. ADP was effective in accelerating the wound closure in diabetic mice compared to the respective saline-treated mice (**Figure 1A**), but did not change the wound healing in non-diabetic mice (**Figure 1B**). ADP-treated diabetic mice presented 60% wound closure versus 2% in saline-treated mice at day 7 (**Figure 1A** - graph). More importantly, the wound closure profile of the diabetic animals treated with ADP was similar to that of saline-treated non-diabetic mice (**Figure 1B** – first row of photographs). Moreover, the only effective dose able to accelerate wound closure was 30 μM (**Figure 1C**-table). Indeed, higher ADP doses tested delayed wound healing when observed at day 14, compared to the saline-treated wound.

### Clop Impairs ADP-Induced Wound Closure

To assess the role of P2Y_12_ and ADP in our model, the P2Y_12_ irreversible antagonist Clop was administrated (5 mg/kg) daily by gavage. This treatment impaired the ADP-mediated wound closure in diabetic mice (**Figure 1A**). It was characterized by an increase of the lesion size and a worsening of the wound general aspects, at all the time-points evaluated. Furthermore, Clop administration also worsened the saline-treated wound of diabetic mice (**Supplementary Figure 1**). Still, an endogenous and physiological critical role in tissue repair of ADP/P2Y_12_ axis was suggested, since Clop treatment also impaired healing of both saline- (**Supplementary Figure 1**) and ADP-treated wounds (**Figure 1B**) of non-diabetic mice. Taking into consideration that ADP is the major agonist of P2Y_12_ receptor (32, 33) and Clop treatment prevented ADP-induced wound closure, these observations provide an unequivocal proof of ADP’s role in accelerating wound closure of diabetic mice.

#### P2Y_1_ Also Seems to Mediate ADP Effects

**Figure 2:**
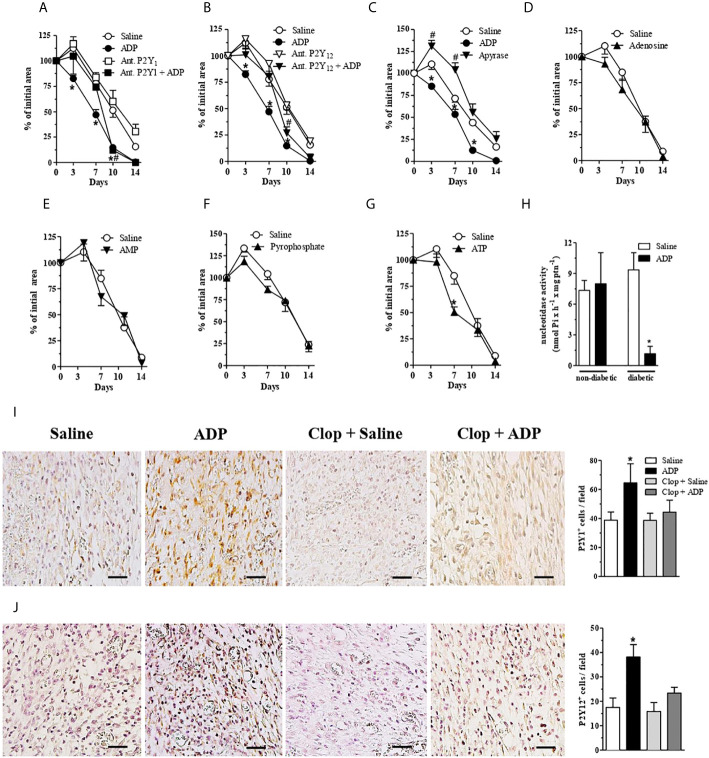
Central role of ADP, P2Y_1_ and P2Y_12_ during wound healing of diabetic mice. **(A, B)** Diabetic mice were subjected to excisional full-thickness wounding and then, topically treated with P2Y_1_ or P2Y_12_ antagonists (30 µM/mouse - 30 µL) 30 min before ADP (30 μM/mouse) or saline administration. Both antagonists were applied every day for 14 days. Open wound area was followed over time as described in **Figure 1**. ^*^P < 0.05 by two-way ANOVA followed by Bonferroni post-test, compared to saline-treated mice; **^#^**P < 0.05 by two-way ANOVA followed by Bonferroni post-test compared between P2Y_1_ or P2Y_12_ antagonists + ADP and saline-treated mice, n=7-10 per group. **(C–G)** Diabetic mice were subjected to excisional full-thickness wounding and then topically treated with apyrase (6 U/mL), ATP, ADP, AMP, adenosine, pyrophosphate (30 µM/mouse - 30 µL) or saline every day for 14 days. Open wound areas were followed over time as described in **Figure 1**. Data are expressed as mean ± standard error of the mean. ^*^P < 0.05 by two-way ANOVA followed by Bonferroni post-test, compared to saline-treated mice; **^#^**P < 0.05 by two-way ANOVA followed by Bonferroni post-test compared between apyrase- and saline-treated mice, n=8-10 per group. **(H)** Non-diabetic and diabetic mice were subjected to excisional full-thickness wounding and then topically treated with ADP (30 µM/mouse) or saline every day for 7 days. The nucleotidase activity was evaluated in the wound tissue harvested at day 7. ^*^P < 0.05 by Student’s t test, compared to saline-treated diabetic mice, n=6 per group. **(I, J)** Photomicrographs and bar graphs of $\text{P}2{\text{Y}}_{1}^{+}$ and $\text{P}2{\text{Y}}_{12}^{+}$ cell numbers per field, respectively. Diabetic mice subjected to excisional full-thickness wounding were topically treated with ADP (30 µM/mouse) or saline every day for 7 days. Both groups were treated by gavage with Clop (5 mg/kg) 1 h before saline or ADP treatment of the wounds. The wound tissues were harvested at day 7 and stained by IHC for P2Y_1_ and P2Y_12_ receptors. Scale bars: 50µm. Data are expressed as mean ± standard error of the mean. ^*^P < 0.05 by one-way ANOVA followed by Tukey post-test, compared to saline-treated mice, n=6 per group.

The involvement of ADP receptors in wound healing was verified by the use of another P2Y_12_ antagonist (MRS2395) and by a P2Y_1_ antagonist (MRS2179). Both antagonists, used at 30 μM (30 μL/wound), impaired the wound closure induced by ADP until day 7. However, at day 10 and 14 the wound healing profile was identical to that observed in ADP-treated group without antagonists administration (**Figures 2A, B**). P2Y_1_ and P2Y_12_ receptor antagonists alone did not accelerate or worsen the wound closure in diabetic mice (**Figures 2A, B**). Since Clop treatment impaired the wound healing in the presence (**Figure 1A**) and absence (**Supplementary Figure 1**) of ADP in diabetic mice, we expected to observe the same response with MSR2395 treatment. Indeed, such divergence could be partially explained by different administration routes (systemic *versus* local) between these drugs; in addition, while Clop is an irreversible antagonist, MRS2395 is a competitive antagonist.

### Apyrase Worsens Wound Healing

Apyrase removes the γ-phosphate from ATP and the β-phosphate from ADP, yielding AMP (34). Apyrase treatment worsened wound healing in diabetic mice, when compared to either saline- or ADP-treated diabetic wounds (**Figure 2C**), confirming the crucial role of this nucleotide in tissue repair.

### Different Nucleotides Do Not Accelerate the Wound Healing

In order to test the effect of other nucleotides in our model, adenosine, AMP, pyrophosphate or ATP were topically applied on the wounds of diabetic mice at 30 μM/mouse, the same optimal concentration previously used for ADP. None of the nucleotides tested improved the wound healing (**Figures 2D–F**), except for ATP treatment, which showed a slight improvement of wound closure at day 7 (**Figure 2G**).

### ADP Reduces Ecto-Nucleotidase Activity in the Wounds of Diabetic Mice

To assess a possible enzyme deregulation related to the metabolism of extracellular ADP during diabetes, the ecto-nucleotidase activity was evaluated in wounds at day 7 after wounding. The enzyme activity detected in the ADP-treated wounds obtained from diabetic mice was reduced compared to saline-treated wounds from diabetic mice, and when compared to wounds from non-diabetic mice (**Figure 2H**). The same profile was observed in blood samples obtained from ADP-treated diabetic mice (data not shown). It seems that ADP treatment downregulates ecto-nucleotidase activity only in diabetic mice, which seems to favor wound healing. Indeed, we did not investigate if the ecto-nucleotidase activity reduction by ADP is due to a decrease in enzyme expression or a direct effect on enzyme activity. Further experiments are necessary to elucidate the mechanism involved.

### ADP Increases P2Y_1_^+^ and P2Y_12_^+^ Cells in the Wounds of Diabetic Mice

ADP-treated diabetic wounds presented higher expression of P2Y_1_ and P2Y_12_ at day 7, when compared to saline-treated wounds (**Figures 2I, J**). Moreover, Clop treatment impaired ADP-induced P2Y_1_ and P2Y_12_ expression, whereas it did not change the expression of such receptors in saline-treated diabetic mice (**Figures 2I, J**). These data suggest that exogenous ADP positively modulates its own response in the wounded skin of diabetic mice.

### ADP Improves Tissue Formation in the Wounds of Diabetic Mice

**Figure 3:**
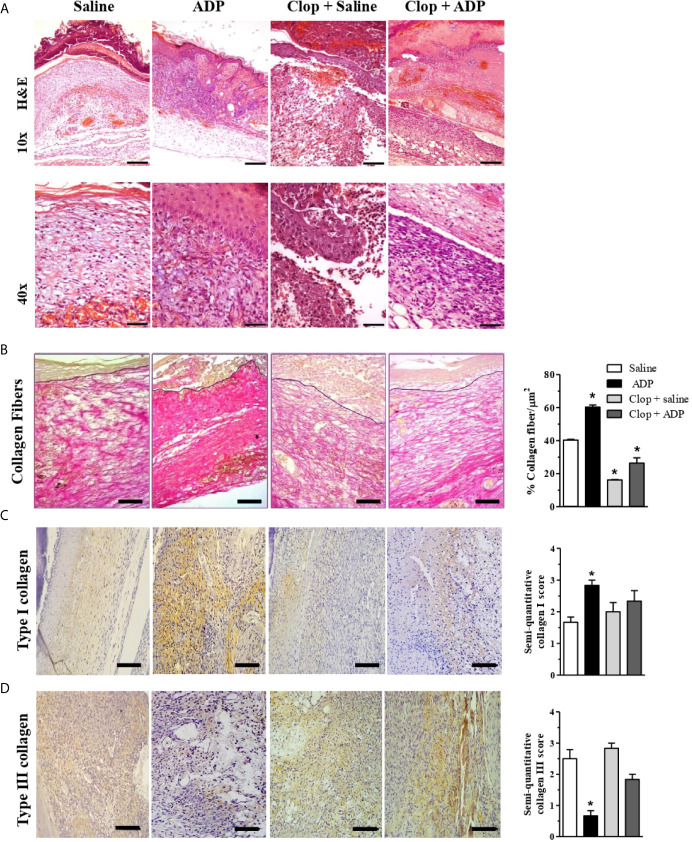
ADP-treated wounds present an improved tissue repair and an increased collagen depo9sition. **(A)** Diabetic mice subjected to excisional full-thickness wounding were treated by gavage with Clop (5 mg/kg) 1 h before ADP (30 µM/mouse - 30 µL) or saline administration, once a day for 7 days. Wounds were harvested at day 7 and stained with hematoxylin and eosin. Representative images of 4-5 mice per group. Scale bars: 10x=200 µm; 20x=100 µm; 40x=50 µm. **(B)** Collagen deposit (red staining) in wounds at day 7 stained with Picro Sirius Red and the representative images are shown; bar graph summarizes data from 5-6 mice per group, representative of three independent experiments. Scale bars: 50 µm. ^*^P < 0.05 by one-way ANOVA followed by Tukey post-test, compared to saline-treated mice. **(C, D)** Type I and type III collagen staining by IHC. Scale bar 50 µm. Graphs with semi-quantification score for type I and type III collagen deposit. Data from one experiment with 3-4 mice per group.

Saline-treated wounds of diabetic mice presented edematous dermis, leukocyte infiltration (predominantly by mononuclear cells), and null (or partial) formation of epidermis at day 7. In the reticular dermis, exuberant formation of granulation tissue and congested neovessels were observed (**Figure 3A**). Interestingly, ADP-treated wounds presented the epidermis regenerated and integrated to the underlying dermis, with hyperplasic suprabasal layers, and hyperkeratosis. In the dermis, there was a dense granulation tissue with inflammatory cell infiltrate comprising eosinophils, mast cells, myeloid progenitors, neutrophils, and mononuclear cells. Clop administration impaired tissue regeneration in saline-treated wounds, where denuded epidermis areas, necrotic dermis with an inflammatory infiltrate composed predominantly of polymorphonuclear cells, striking bleeding, and the absence of granulation tissue were observed. Clop administration also impaired the tissue formation in ADP-treated mice, however, performing milder effects. In this case, wounds displayed a more organized reticular dermis, with collagen bundles parallel to the skin surface and interspersed with fibroblasts; a few vessels and inflammatory infiltrate were also noticed (**Figure 3A**).

Picro Sirius Red stained tissue photomicrographs (red staining) showed higher deposition of collagen fibers in ADP-treated wounds of diabetic animals compared to the saline-treated wounds (**Figure 3B**). Clop administration impaired collagen deposit in both ADP and saline-treated wounds. Collagen fibers quantification confirmed that ADP treatment enhanced collagen deposition while Clop administration impaired its accumulation (**Figure 3B**-graph). ADP seemed to accelerate the switch of type III to type I collagen, a more mature fiber (**Figures 3C, D**). Nevertheless, Clop administration reduced type I collagen deposit, without changing type III collagen deposit in both saline- and ADP-treated wounds. The results depicted in the bar graphs represent the photomicrographs (**Figures 3C, D**-graphs).

### ADP Induces Keratinocyte Proliferation in Diabetic Wounds

**Figure 4:**
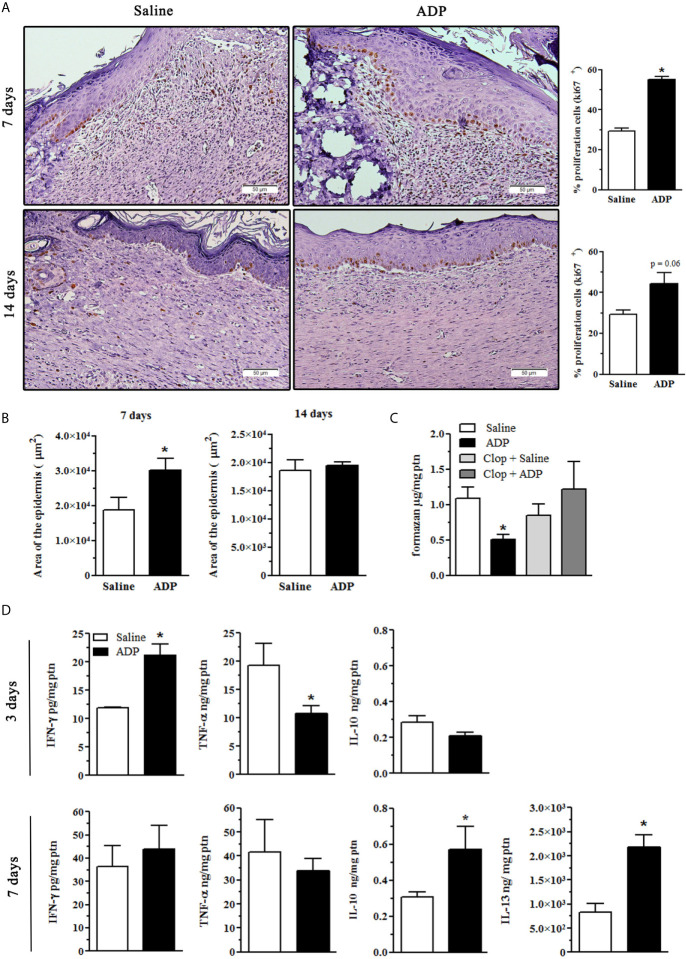
ADP induces keratinocyte proliferation and modulates cytokine and free-radical production. Diabetic mice were subjected to excisional full-thickness wounding, and then, topically treated with ADP (30 µM/mouse - 30 µL) or saline every day for up to 14 days. **(A, B)** Wound tissues were harvested from diabetic mice at days 7 and 14 after wounding, and stained for Ki67 by IHC and with hematoxylin and eosin. Scale bar: 50 µm. The percentage of proliferating keratinocytes (Ki67^+^) and the area of epidermis were represented in bar graphs. Data are expressed as mean ± standard error of the mean. ^*^P < 0.001 by Student’s t test, compared to saline-treated mice; n= 6 per group. **(C)** Superoxide radical production was indirectly evaluated in the wounds obtained at day 7 after wounding by f9ormazan generation as final product. Some animals were treated by gavage with Clop (5 mg/kg) 1 h before ADP or saline wound topic treatment. ^*^P < 0.05 by one-way ANOVA followed by Tukey post-test, compared to saline-treated mice, n=4-5 per group. **(D)** Wound tissues were harvested from diabetic mice at days 3 and 7 after wounding and cytokine levels were evaluated by ELISA. ^*^P < 0.05 by Student’s t test, compared to saline-treated mice, n=4-6 per group.

We next evaluated if ADP enhances re-epithelization. At day 7 after wounding, ADP-treated wounds presented a higher number of cells expressing Ki67, a cell proliferation marker, in the layer adjacent to the basal membrane when compared to saline-treated wounds in diabetic mice. At day 14, the frequency of Ki67^+^ cells was still higher than that of saline-treated wounds, but to a lesser extent (**Figure 4A**). The percentage of proliferating cells, observed at days 7 and 14 post wounding, is shown as bar charts (**Figure 4A**-graphs). Corroborating this result, epidermis area was also larger in ADP-treated wounds at day 7 compared to saline-treated wounds, while at day 14 it returned to normal (**Figure 4B**).

### ADP Modulates the Inflammatory Response

We observed a reduction in the production of reactive oxygen species at day 7 post-wounding after in ADP-treated mice, while Clop administration restored reactive oxygen species production (**Figure 4C**), suggesting again the participation of P2Y_12_. At day 3 after wounding, ADP treatment promoted an increase of IFN-γ and a reduction of TNF-α levels without affecting IL-10 levels, while increased IL-10 and IL-13 levels were observed at day 7 (**Figure 4D**). No differences were detected in IL-6, IL-12p70 and CCL2 levels between the groups (data not shown). These results suggest that ADP treatment controls inflammatory response associated with pro-resolution effects.

### ADP Increases Myofibroblasts Population and Transforming Growth Factor- β (TGF-β) Production

**Figure 5:**
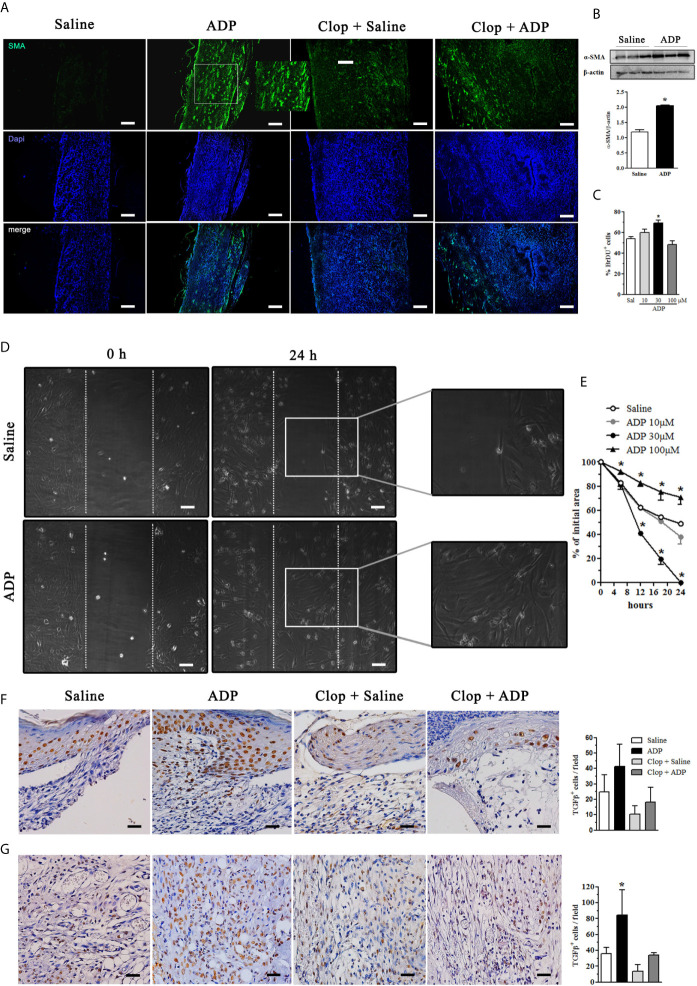
ADP activates myofibroblasts/fibroblasts and increases the amount of TGF-β^+^cells in the wounds of diabetic mice. Diabetic mice were subjected to excisional full-thickness wounding and then, topically treated with ADP (30 µM/mouse - 30 µL) or saline every day for 7 days. Some mice were treated by gavage with Clop (5 mg/kg) 1 h before ADP or saline administration, once a day for 7 days. **(A)** Wound tissues harvested at day 7 were stained for α-SMA (green) and DAPI (blue) and analyzed by immunofluorescence. **(B)** Gel bands and graphs depicting the semi-quantification of α-SMA by WB. Each bar represents a pool of skin-derived protein extracts obtained from at least 5 mice. ^*^P<0.05 by Student’s t test compared to saline-treated mice; data are representative of two independent experiments. **(C)** Primary culture of neonate murine dermal fibroblasts was plated for 24 h, incubated with BrdU for more 24 h and the cell proliferation was evaluated by immunofluorescence. ^*^P<0.05 by one-way ANOVA followed by Tukey post-test, compared to saline-treated mice; data are representative of three independent experiments. **(D, E)** Primary dermal murine fibroblasts were plated for 24 h, pre-incubated with mitomycin-C 5 μg/mL for 2 h and then incubated with different concentrations of ADP. The open ar9ea between the front edges of the scratch were evaluated at 0, 6, 12, 18 and 24 h after scratch and expressed as % of initial area. Fibroblast culture images represent only the first and last time points evaluated for cell migration. ^*^P < 0.05 by two-way ANOVA followed by Bonferroni post-test, compared to saline-treated mice, the data are representative of three independent experiments. Photomicrographs and bar graphs of TGF-β^+^ cells determined by IHC in the **(F)** epidermis and **(G)** dermis obtained at day 7 after wounding. ^*^P < 0.05 by one-way ANOVA followed by Tukey post-test, compared to saline-treated mice, n=8 per group.

Myofibroblasts present high ability of extracellular matrix protein production and wound contraction (1). We observed that ADP treatment increased α-SMA expression in the dermis, which was reduced by Clop; in the Clop + ADP group a less dramatic reduction of myofibroblasts was observed by immunofluorescence (**Figure 5A**). The ADP-induced α-SMA expression in diabetic wound was confirmed by WB analysis (**Figure 5B**). In accordance, ADP at 30 µM (but not at other concentrations tested) also induced proliferation of murine fibroblasts *in vitro* (**Figure 5C**). In addition, fibroblasts treated with ADP at 30 µM presented a better migration capacity when compared to saline-treated cells or with cells treated with ADP at 10 and 100 µM, suggesting a dose-dependent effect of ADP on wound healing, in accordance with earlier data (**Figures 5D, E**).

TGF-β is a pivotal cytokine that regulates myofibroblast differentiation and activation, re-epithelization, and activation of alternative macrophages, which are essential steps for wound healing (1, 2). ADP treatment seemed to increase TGF-β production by keratinocytes in the epidermis (**Figure 5F**) and the number of TGF-β^+^ cells in the dermis (**Figure 5G**). These results reinforce the pro-resolution role of ADP in wound healing.

### ADP-Treated Wounds Present a Different Leukocyte Profile

**Figure 6:**
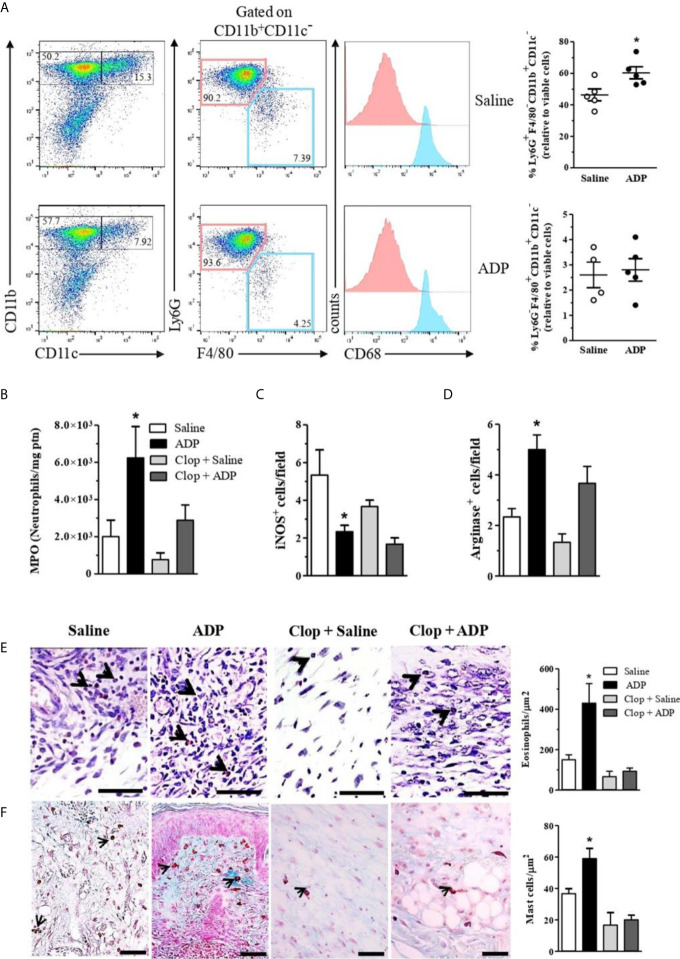
ADP-treated wounds present an increase of neutrophils, arginine^+^ cells, eosinophils and mast cells. Diabetic mice were subjected to excisional full-thickness wounding and then, topically treated with ADP (30 µM/mouse - 30 µL) or saline every day for 7 days. Some mice were treated by gavage with Clop (5 mg/kg) 1 h before ADP or saline administration, once a day for 7 days. Wound tissues were harvested at day 7 after wounding and cell suspensions were analyzed by flow cytometry. **(A)** Dot-plots (left) and graphs (right) show Ly6G^+^F4/80^-^ and Ly6G^-^F4/80^+^ populations (gated on live CD11b^+^CD11c^-^ cells) in the wounded skin. Graphs show the frequency of each cell population relative to gated live cells; for that, percentages of Ly6G^+^F4/80^-^ [neutrophils – depicted in pink] or Ly6G^-^F4/80^+^ [macrophages – depicted in blue] cells were multiplied by the percentage of live CD11b^+^CD11c^-^ cells; intracellular CD68 staining confirms macrophage identity; data representative of one experiment with n=4-5 mice per group; *P < 0.05 by Studen9t’s t test compared to saline-treated mice **(B)** Wound tissues were harvested at day 7 and prepared for myeloperoxidase quantification; and bar graphs are representative of three independent experiments with n=6 per group; **(C)** Wound tissues were evaluated by IHC for inducible nitric oxide synthase^+^ or **(D)** arginase^+^ cells at day 7 after wounding. *P < 0.05 by one-way ANOVA followed by Tukey post-test, data are representative of two independent experiments with n=6 per group. Skin histological sections of wound tissues harvested at day 7 and stained with **(E)** modified Sirius Red stain for eosinophil or with **(F)** Alcian Blue stain for mast cells. Bar graphs represent the number of eosinophils or mast cells per µm^2^. Scale bars=50µm. ^*^P < 0.05 by one-way ANOVA followed by Tukey post-test, compared to saline-treated mice. Data are representative of one experiment with 5-6 mice per group.

The presence and involvement of inflammatory and immune cells in wound healing are well described (35). Unbalanced numbers and/or activation of local leukocytes are common in diabetes, which compromises tissue repair (36). Interestingly, we observed an increase of neutrophil (CD11b^+^CD11c^-^Ly6G^+^F4/80^-^CD68^-^ cell population, indicated in pink) recruitment in ADP-treated wounds by flow cytometry (**Figure 6A** - upper graph). Consistent with that, the enhancement of myeloperoxidase (MPO) activity seen in ADP-treated wounds, relative to saline-treated wounds, was significantly reduced by Clop administration (**Figure 6B**). In parallel, a decrease in the inducible nitric oxide synthase^+^ cells and an increase in the arginase^+^ cells were detected in the ADP-treated wounds (**Figures 6C, D**). This result suggests that monocyte/macrophage population (CD11b^+^CD11c^-^Ly6G^-^F4/80^+^CD68^+^, indicated in blue) may have switched towards an alternatively-activated phenotype, since its frequency was similar between groups (**Figure 6A** - bottom graph). Clop treatment prevented the ADP-induced change of macrophage phenotype in the wound (**Figures 6C, D**). Therefore, our data suggest an ADP-mediated skewed response towards pro-resolution scenario in the context of tissue injury.

Moreover, histological examination of the skin sections from ADP-treated wounds showed increased number of eosinophils (**Figure 6E**) and mast cells (**Figure 6F**) compared to saline-treated wounds. Clop administration did not modify eosinophil and mast cell populations in the saline-treated wounds, although impairs ADP-induced accumulation of both cell types, indicating P2Y_12_ involvement in their recruitment and/or survival.

**Figure 7:**
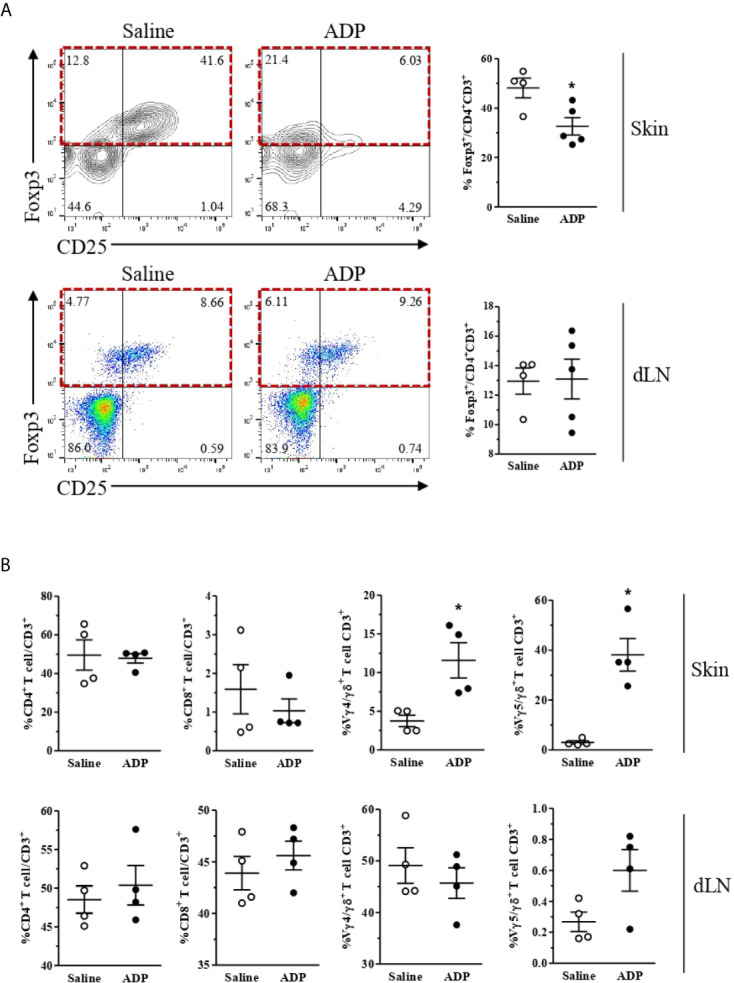
ADP-treated wounds present a reduced population of Tregs and an increase of Vγ4 and Vγ5 T cells. Diabetic mice were subjected to excisional full-thickness wounding and then topically treated with ADP (30 µM/mouse – 30 μL) or saline every day for 7 days. Wound tissues and the skin-draining lymph nodes were harvested at day 7 after wounding and cell suspensions were analyzed by flow cytometry. **(A)** Contour-plots (top left), dot-plots (bottom left), and respective graphs (right) show the frequencies of Foxp3^+^Tregs (relative to CD4^+^CD3^+^population) in the skin and draining lymph nodes (dLN); **(B)** CD4^+^ 9and CD8^+^ T cells (relative to total CD3^+^ lymphocytes), Vγ4^+^, and Vγ5^+^ cells (relative to total γδ^+^T lymphocytes) in the skin and dLN. Data were expressed as mean ± standard error of the mean. *P < 0.05 by Student’s t test compared to saline-treated mice; n=4-5 per group, data are representative of two independent experiments, except for the γδ^+^ T lymphocyte data, which represent one experiment.

T cells are resident in normal human and mouse skin and participate in cutaneous immunosurveillance, contributing to skin homeostasis (36, 37). Thus, we evaluated T cell profile in the skin and wound-draining lymph nodes of diabetic mice after ADP treatment. Interestingly, the percentage of Tregs (forkhead box protein P3 [FoxP3^+^]/CD4^+^CD3^+^) was selectively reduced in the ADP-treated wounds relative to saline-treated wounds, but not in the draining lymph nodes (**Figure 7A**).

In parallel, ADP did not alter CD4^+^ and CD8^+^ T cells frequencies in the skin and lymph nodes; however, ADP-treated wounds showed increased proportions of skin-associated gamma delta (γδ) T cells subtypes as Vγ4^+^ and Vγ5^+^ (**Figure 7B**). Again, no changes in overall T cell populations were seen in the draining lymph nodes after wounding.

### ADP Does Not Improve Wound Healing of Cutaneous Ulcer Induced by *Leishmania amazonensis*

**Figure 8:**
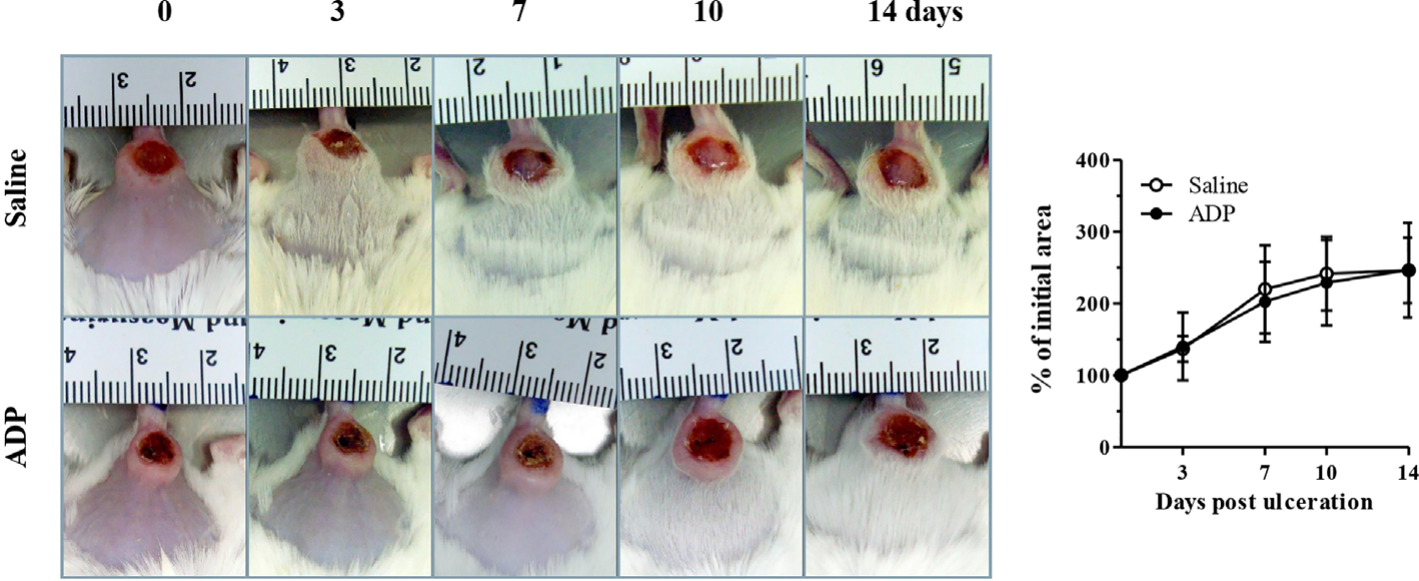
ADP did not accelerate wound healing of cutaneous leishmaniasis. BALB/c mice were intradermally inoculated with 10^6^ promastigote/mouse/50 μL (2×10^8^/mL). After wound ulceration, animals were topically treated every day with saline or ADP (30 µM/mouse – 30 μL) per 10 days. Open wound area was measured at days 0, 3, 7, 10, and 14. Representative images and graph of cutaneous leishmaniasis-associated wound treated with saline or ADP. The areas at day 0 were considered 100%, and the subsequent areas were proportional (%) to the initial wound area; n=7 per group.

We also evaluated the effect of ADP on cutaneous ulcer induced by experimental *Leishmania amazonensis* infection and no improvement was observed (**Figure 8**). These results indicate that ADP may be context-dependent and possibly effective just in wounds of individuals with metabolic diseases such as diabetes.

## Discussion

In this paper, we provide the first evidence that ADP plays a pivotal role as a potent agent that accelerates cutaneous wound healing in diabetic mice. Due to the large number of patients suffering from diabetes worldwide that present a poor quality of life and high risk of complications as chronic wounds, we emphasize the importance of a better comprehension of the pathophysiology and the mediators involved in wound healing.

ADP is an endogenous nucleotide which acts as a potent mediator in platelet aggregation and inflammation, being quickly metabolized. The role of ADP in tissue repair has was always been related to platelet aggregation, since ADP is rapidly released from activated platelets, acting in an autocrine way together with histamine, serotonin, calcium and several other mediators for platelet aggregation, driving the return to homeostasis (38). Besides, other authors have previously described an effect of ADP on cell proliferation, such as on cell culture of murine fibroblasts (3T3 and 3T6), isolated rat chondrocytes and zebrafish retinal cells (14, 15, 39, 40), suggesting that ADP may improve healing and regeneration due to these properties. Corroborating with literature data, nucleotides, including ADP, induce epithelial cell migration in an *in vitro* model of wound healing using a quiescent monkey kidney epithelial culture (41). Similar data was observed in a study with non-transformed small intestine epithelial cell line IEC-6 using the same *in vitro* wound model, where ADP and ATP stimulate epithelial migration (42).

Given the above findings, we hypothesized that ADP modulates many other molecular and cellular aspects of inflammation during tissue repair, promoting an efficient wound healing. Therefore, our study reports an important role of exogenous ADP in stimulating immune cells activation, resolution of inflammation, and restoration of tissue integrity in non-healing wounds of diabetic mice.

Controlled inflammation is one of the major steps for wound healing. The absence of inflammatory response or its exaggerated activation impair the natural progression of wound healing towards the proliferative and remodeling/healing phases. An unbalanced ADP production/action, which is observed in diabetes, could impact on inflammation process. Thus, exogenous ADP seems to adequately modulate this process in our model, recovering the normal evolution of healing. Therefore, it is reasonable to observe that, when topically applied during the initial healing phase, ADP just improved the wound healing of diabetic mice but not of healthy non-diabetic animals, which are already extremely competent in tissue repair. Of note, ADP-treated wounds in diabetic mice heal at the same rate seen in wounds of non-diabetic mice. Interestingly, we found that a 5-day treatment protocol with ADP was also effective in accelerating wound healing (data not shown) as observed to the 14-days treatment. This result support the role of ADP in the initial healing steps.

Taking these data into account, we suggested a possible failure in the nucleotide pathway during diabetes. Thus, in an attempt to explain this phenomenon, we raised some possibilities such as: (i) deficient ADP production; (ii) upregulation of ADP degradation by ecto-nucleotidases; (iii) inefficient expression/activation of purinergic receptors in the skin of diabetic mice. First, we addressed ADP production in the skin by HPLC, however we found low levels of ADP in the wounds since it is a liable molecule that is metabolized in less than 5 minutes, which makes its quantification unfeasible (data not shown). Second, regarding the enzymes that degrade extracellular nucleotides, the ecto-nucleoside triphosphate diphosphohydrolase-1 (CD39) hydrolyses extracellular ATP and ADP into AMP, which is subsequently converted to adenosine (ADO) by the action of ecto-5’-nucleotidase (CD73) (43, 44). Previous studies have demonstrated that the nucleotidase activity is increased in diabetic patients and associated pathologies. Moreover, hydrolysis of adenine nucleotides is increased in platelets from diabetic patients (45), which can partly explain beneficial effect exogenous ADP. In our data, exogenous ADP treatment inhibited overall nucleotidase activity in the wound of diabetic animals, favoring the ADP effect in wound healing. Lastly, the increased expression of P2Y_1_ and P2Y_12_ receptors in the diabetic wound observed after ADP treatment suggests a possible deficiency in the expression of nucleotide receptors during diabetes. Altogether, part of the beneficial effects of ADP on diabetic wounds appear to be by up regulating its own receptors in the skin and by reducing the nucleotidase activity.

We explored several strategies in order to demonstrate the major role of ADP on wound healing in diabetic mice. Initially, we demonstrated that ATP, AMP, ADO, and pyrophosphate were not as effective as ADP at the low concentration of 30 μM. Furthermore, the role of ADP in our system was also confirmed using apyrase, enzyme responsible for degrading ATP and ADP into AMP. In fact, apyrase administration worsened wound healing of diabetic mice, excluding at the same time the role of AMP accumulation derived from ADP degradation as a possible mechanism for ADP-induced tissue repair.

ADP poses several advantages among other nucleotides. Studies already demonstrated the role of ADO and its receptors in wound healing. For instance, daily treatment of healthy and diabetic rats with A2A receptor agonist accelerated wound healing by stimulating fibroblast and endothelial cell migration to the injured area and by reducing inflammation (46, 47). Surprisingly, Montesinos et al. (47), using A2A receptor knockout mice, also demonstrated the importance of A2A receptor to the formation of uniform granulation tissue and angiogenesis (47). ATP was also described to accelerate wound healing. ATP-containing vesicles promote a massive influx and *in situ* proliferation of macrophages, the release of pro-inflammatory cytokines and vascular endothelial growth factor, neovascularization and collagen production (48). Meanwhile, it is important to stress that ATP directly excites primary sensory neurons, triggering pain signaling, a fact that undermines its therapeutically use for wound healing (49). Also, it was already described that *Staphylococcus aureus* USA300, one of the most prevalent bacterial species identified in chronic wounds, exploits the immunomodulatory characteristics of ADO to subvert host immune response (50), retarding the healing of infected wounds. Lastly, ADO and ATP also promote fibrosis when released in high concentrations or chronically (51–53), which denotes a negative aspect in the case of topically application.

To confirm our findings, we used an ADP receptor antagonist. Clop is a prodrug used widely as a platelet aggregation inhibitor and exerts its action by irreversibly antagonizing the P2Y_12_ receptor (54). It is worthy to state that ADP is the major ligand of P2Y_12_ receptor (31, 32). Our study showed that Clop impaired the effect of exogenous ADP in diabetic wounds, and more strikingly, also in non-diabetic mice, which revealed the role of endogenous ADP and P2Y_12_ receptor in tissue repair. Similarly, the same profile was observed with a P2Y_1_R antagonist (MRS2179) and a different P2Y_12_R antagonist (MRS2395), confirming the role of both receptors in wound healing. Remarkably, the effect of those ADP receptor antagonists was lost after day 7. One explanation for that could be an increase in tissue sensitivity due to topic ADP application, since we already demonstrated an ADP-driven up regulation of P2Y_1_ and P2Y_12_ receptors, which may be due to a recruitment of purinergic receptor-expressing cells to the tissue or by an up regulation of those receptors by resident cells. We could also not discard a possible involvement of P2Y_13_ receptor in the final stage of wound healing, although unfortunately the P2Y_13_ antagonist was not available.

The role of ADP in wound healing is expected since P2Y_1_, P2Y_12_, and P2Y_13_ receptors are expressed in all kinds of cell types important for tissue repair (leukocytes, endothelial cells, keratinocytes, fibroblasts, and platelets). They are involved in cell activation, migration, and proliferation (12, 31, 35). However, the exact effects of these receptors and ADP in wound healing are not well determined. A successful healing results in the reconstitution of skin tension, resistance, and function to a similar degree as those of the original tissue, relying on (i) deposition of extracellular matrix proteins, such as collagen; (ii) formation of basal membrane, epidermis and new vessels, and (iii) repopulation of resident cells (1). Here, we showed that ADP improved the tissue formation by promoting less edema, accelerated re-epithelization, increased cell infiltration, and collagen deposit. ADP-treated wounds were characterized by a significant cell migration, such as leukocytes and fibroblasts, from the edge towards the center of the lesion. Note that the arrival of these cells marks the formation of granulation tissue, which is crucial for the healing process. These results suggest that ADP acts as a pro-inflammatory and pro-resolution molecule, providing a tissue formation of superior quality and organization compared to that observed in untreated wounds of diabetic mice. In rats, P2Y_1_ receptor is expressed by cells of the basal layer, which is the site of keratinocyte proliferation (55). Yoshida et al. (56), demonstrated mRNA expression for P2Y_1_ e P2Y_12_ receptors in a culture of keratinocytes, whereas others reported that fibroblasts express P2Y_1_, P2Y_12_, and P2Y_13_ receptors (49, 56, 57). Thus, ADP receptors are widely expressed in the skin corroborating the pleiotropic effect of ADP in wound healing during diabetes.

Fibroblasts/myofibroblasts are cells that approach the edges of the wounds and produce extracellular matrix, primarily collagen, which is the major component of the mature scar (58). An increase in the number of myofibroblasts induced by ADP treatment helps to explain the accelerated tissue repair, the increase in the collagen deposit, and its correlation with the increase of TGF-β^+^ cells in the dermis and epidermis. Again, by inhibiting P2Y_12_ receptor several parameters were reduced in the injured skin of diabetic mice, including myofibroblast differentiation/activation, TGF-β production and collagen deposit, which in turn diminished granulation tissue formation, resulting in impairment of wound healing. Moreover, the shift from type III to type I collagen, triggered by ADP treatment, provided a more mature connective tissue and scar. Type I collagen is the most abundant collagen type in health skin and is associated with scar maturation (59, 60). The positive effect of ADP on fibroblast was also confirmed by *in vitro* experiments, since this nucleotide induced fibroblast proliferation and migration.

The balance of pro- and anti-inflammatory cytokines is essential for successful healing, while an overwhelming cytokine production as well as no production impair wound healing (61). Of note, Lin et al. (62), demonstrated that IL*-*6 knockout mice present a delay in the wound closure, a reduction in leukocyte infiltration, re-epithelialization, angiogenesis, and collagen deposition, compared to wild type mice (62). Others demonstrated that TNF^-/-^ mice have a better granulation tissue formation but a compromised re-epithelialization (63). Also, CCL2 knockout mice exhibit a delay in re-epithelialization, angiogenesis, and collagen synthesis (64). In our analyzes, the increased levels of IFN-γ at day 3 and an increase of IL-10 and IL-13 at day 7 after wounding suggest an anticipation in the shift of inflammatory to resolution phase induced by ADP treatment. The increased amount of TGF-β in the skin after ADP treatment supports the idea of transition to an earlier resolution phase (65).

An intense inflammatory infiltrate in the wound tissues was observed after ADP application. It is noteworthy that the inflammatory process during normal wound healing is characterized by spatial and temporal changes in leukocytes’ patterns. The well-defined chronology of these events is essential for ideal repair (66). Tissue macrophages are activated by IL-4 and IL-13 cytokines and converted in a cell-type programmed to promote wound healing (67). The high concentration of IL-13 at day 7 together with a shift of macrophage phenotype from M1 to M2 after ADP treatment corroborates with our hypothesis that ADP is a pro-resolution molecule. Similarly, mast cells are skin-resident cells that accumulate and are necessary for wound healing (68). However, according to the literature mast cells can favor or impair wound healing, depending on the stimulus intensity (69). In our diabetes model, the increased mast cell population after ADP treatment suggests a positive role in wound healing, but other experiments must be done to confirm this correlation.

Studies demonstrated that neutrophils, isolated from wound sites, can also regulate the innate immune response during healing (66). Zhang et al. (70), showed that neutrophils have a regulatory role in the inflammatory response by secreting IL-10 (70). The neutrophil accumulation in the wound after ADP treatment at day 7, together with increased IL-10 levels at the same time-point supports our hypothesis that ADP drives the neutrophil activation to a tissue-repair profile. Another polymorphonuclear cell type, the eosinophil, also infiltrates into wounds (often in close proximity with fibroblasts), stores TGF-β and seems to release it, as demonstrated in a rabbit cutaneous open wound model (71, 72). Therefore, the eosinophils recruited by ADP at day 7 may contribute to accelerate the wound healing *via* TGF-β production and collagen deposition. Here we demonstrated that ADP promoted an exuberant accumulation of eosinophils within wounds of diabetic mice, which correlated with improved tissue recovery.

Skin also hosts αβ and γδ T lymphocytes, which maintain tissue homeostasis by modulating keratinocyte differentiation (re-epithelization), responding to infection, and regulating wound repair. A balance between Tregs, Th17 cells, and γδ T cells plays an important role in skin homeostasis (35). The reduction of Tregs and increase of Vγ4^+^ and Vγ5^+^ cells (two γδ T cells subtypes) in the skin after ADP treatment provide evidence for recovery of epidermal barrier function, as well as of the innate immune response. Strikingly, mice deficient for γδ T cells, including dendritic epidermal T cells (which express the Vγ5^+^ TCR), present a delay in wound healing and a defect in their ability to clear intradermal *S. aureus* infection (73). This type of cell produces insulin growth factor-1 and keratinocyte growth factor-2, molecules related to re-epithelization and skin wound repair (74). Vγ4^+^ cells migrate to murine dermis and epidermis after wounding and are the major source of IL-17A, which in turn enhances neutrophil migration and induces IL-1 and IL-23 production from epidermal cells to initiate local inflammation, necessary for wound healing (75).

Finally, our findings suggest that ADP has beneficial effects mainly in diabetic wounds, since ADP did not accelerate wound healing neither in naive mice nor in *Leishmania amazonensis-*induced skin lesion.

**Figure 9:**
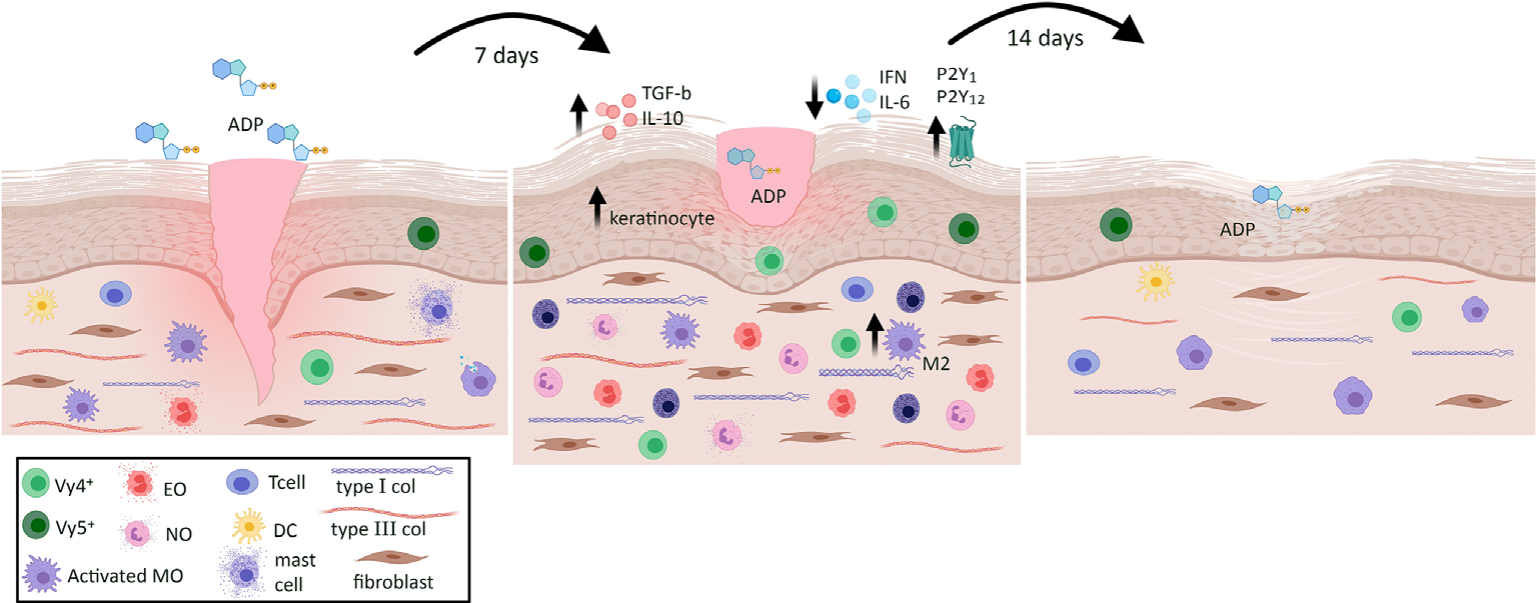
Summary of the pleiotropic effects of ADP on skin wound in diabetic mice. ADP topical instillation accelerates wound closure and improves tissue repair represented by type I collagen deposit and adequate reepithelization. The mechanisms seem to involve the increase of neutrophils, eosinophils, mast cells, M2 macrophages, myofibroblasts and Vγ4^+^ and Vγ5^+^ cells in the wound, besides its ability in modulating cytokine release. ADP plays pivotal role within inflammation, proliferation and remodeling phases during skin tissue repair in diabetes wounds.

In conclusion, we provide novel insights into the pathophysiology of wounds that are difficult to heal, as well as the crucial role of endogenous and exogenous ADP on tissue repair. The real mechanism underlying the effect of ADP on wound repair in diabetes is not completely understood, but we provide the evidence that ADP promotes skin homeostasis by inducing a brief and balanced inflammatory process and by recruiting and/or activating immune cells, followed by a switch to adequate proliferation and remodeling phases (**Figure 9**). Still, another point to be raised and that deserves caution is related to Clop, since our data also brings out a potential harmful effect for thrombosis patients who have wounds.

## Data Availability Statement

The raw data supporting the conclusions of this article will be made available by the authors, without undue reservation.

## Ethics Statement

The animal study was reviewed and approved by Ethics Committee for the Use of Animals of the Federal University of Rio de Janeiro, protocol number 093/15.

## Author Contributions

PB, JG and IW: conceptualization, formal analysis, investigation, methodology, writing–original draft, and editing. JB, VF-J, FL, and TR-A: collected and analyzed the data for this work. ES: designed and performed the Leishmania experiment. CT: performed and analyzed the histology data. RC-S and JM-F: formal analysis and investigation of purinergic biology. CP: conceptualization formal analysis of γδ T cells assay. JN and PM: conceived the project. FC: performed and analyzed all FACs data. CM: performed and analyzed IF data. CC: investigation, writing–review and editing. CB: conceived the project, formal analysis, investigation, project administration, resources, supervision, writing–original draft, writing–review and editing. All authors contributed to the article and approved the submitted version.

## Funding

This work was supported by grants from National Council for Scientific and Technological Development (Conselho Nacional de Desenvolvimento Científico e Tecnológico - CNPq), Research Support Foundation of the State of Rio de Janeiro (Fundação de Amparo à Pesquisa do Estado do Rio de Janeiro – FAPERJ), Coordination of the Improvement of Higher Education Personnel – (Coordenação de Aperfeiçoamento de Pessoal de Nível Superior CAPES) and Health Ministry.

## Conflict of Interest

The authors declare that the research was conducted in the absence of any commercial or financial relationships that could be construed as a potential conflict of interest.

## Abbreviations

ADO, adenosine; ADP, adenosine diphosphate; ATP, adenosine triphosphate; AMP, adenosine monophosphate; BrdU, 5-bromo-2’-deoxyuridine; CBA, cytometric bead array; CCL2, C-C motif chemokine ligand 2; Clop, clopidogrel; DAPI, 4’,6-diamidino-2-phenylindole; FACS – Foxp3- forkhead box protein p3; gamma delta T cells, γδ T cells; IFN, interferon; IHC, immunohistochemistry; IL, interleukin; PBS, phosphate buffer saline; ROS, reactive oxygen species; α-SMA, α-smooth muscle actin; TGF-β, transforming growth factor-β; TNF-α, tumor necrosis factor-α; Tregs, regulatory T cells; WB, western blotting.
